# Use of Biomaterials for 3D Printing by Fused Deposition Modeling Technique: A Review

**DOI:** 10.3389/fchem.2020.00315

**Published:** 2020-05-07

**Authors:** Sanjita Wasti, Sushil Adhikari

**Affiliations:** Department of Biosystems Engineering, Center for Bioenergy and Bioproducts, Auburn University, Auburn, AL, United States

**Keywords:** 3D printing, fused deposition modeling, biomaterials, biopolymers, composites, biofillers

## Abstract

Three-dimensional (3D) printing is a revolutionary manufacturing technique that can fabricate a 3D object by depositing materials layer by layer. Different materials such as metals, polymers, and concretes are generally used for 3D printing. In order to make 3D printing sustainable, researchers are working on the use of different bioderived materials for 3D printing. Because of the abundant and sustainable sources, and versatile properties, biomaterials are considered as the potential candidates that have the ability to replace petroleum-based polymers. This review highlights the basic overview of fused deposition modeling (FDM) technique of 3D printing and recent developments that have occurred on FDM printing using biomaterials. Specifically, FDM printing process, final properties, and characteristics of biopolymers, their composites, and polymers containing biofillers are discussed.

## Introduction

**Figure 1:**
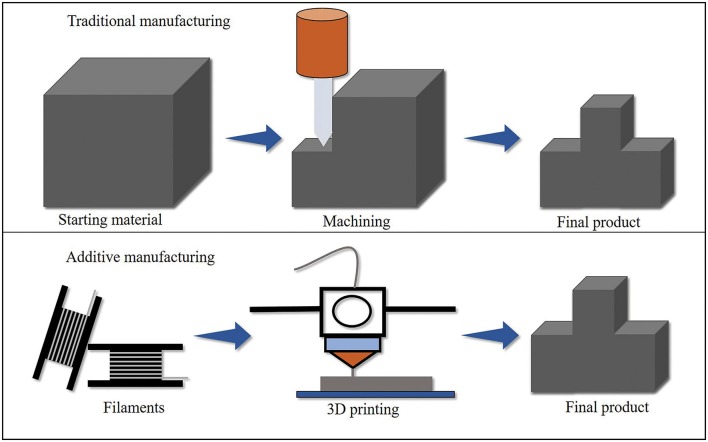
Conceptual comparison of traditional and additive manufacturing (Persons, 2015).

Three-dimensional (3D) printing, also known as additive manufacturing, is transforming manufacturing technology at an amazing rate. It is an emerging technology implemented in different sectors such as research, automotive, aerospace, healthcare and medical, architecture and construction, fashion industries, and food industries (Melnikova et al., 2014; Perkins and Skitmore, 2015; Ozbolat et al., 2016; Shishkovsky, 2016; Wu et al., 2016; Chiulan et al., 2017; Liu et al., 2017; Tao et al., 2017; Vanderploeg et al., 2017; Grimmelsmann et al., 2018; Liu J. et al., 2019). Interest in 3D printing has greatly increased since 2013 and is expected to grow from $6 billion in 2016 to $21 billion by 2021 (Chiulan et al., 2017) due to its unique advantages such as freeform fabrication, sustainable and efficient manufacturing, and shorter time from design to production as compared to subtractive or traditional manufacturing technology (Meteyer et al., 2014; Wimmer et al., 2015; Ou-Yang et al., 2018). In traditional manufacturing such as milling, grinding, and machining, products are fabricated by removing materials from large stock or sheet that may not be able to meet the requirement of small and highly complex products. This drawback of traditional manufacturing is overcome by 3D printing process as it fabricates highly complex parts by adding the materials layer by layer with minimum waste. Contrary to other traditional techniques such as injection molding and compression molding, 3D printing process does not require molds for producing parts, which results in time and cost saving (Ecker et al., 2017). Even after having many advantages over traditional manufacturing, poor mechanical properties, anisotropic nature of printed parts, and limited availability of materials limit its application in large scale and various industries (Ngo et al., 2018). Figure 1 shows the conceptual comparison between traditional and additive manufacturing processes.

**Figure 2:**
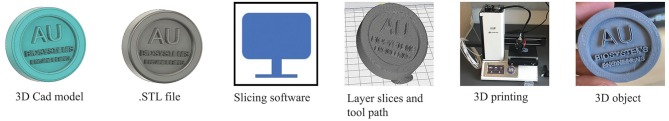
Process of 3D printing (Campbell et al., 2011; Wimmer et al., 2015).

Figure 2 depicts the steps during the 3D additive manufacturing process. The first step of 3D printing is creating a 3D object in Computer-Aided Design (CAD) software and converting it into the standard format of STL (Standard Tessellation Language). This file is then used in slicing software that slices the object in different layers. We can also change different printing parameters such as the material deposition plane, the number of envelopes of the parts, and their thickness and filling patterns. The file obtained from the slicing software is then used in a printer to print the final object.

The first 3D printing process developed was stereolithography (SLA) by Charles Hull in 1986 (Ngo et al., 2018). To date, there are many 3D printing processes that have been categorized into seven major groups by the American Society for Testing and Materials (ASTM) as shown in Table 1 (ASTM International, 2013; Lee et al., 2017). Table 1 presents the seven categories of 3D printing processes along with their brief description, different technologies under each category, materials used, and pros and cons of each category.

**Table 1:** AM process category (ASTM International, 2013; Baumers et al., 2017; Kellens et al., 2017; Lee et al., 2017).

**Process category**	**Description**	**Technologies**	**Materials**	**Pros**	**Cons**
Binder jetting	Inorganic or organic binders are used to bind powder materials	BJ, PBIH, PP	Polymers, metals, sand, biobased materials	Variety of materials can be used, high precision, colored parts	Requires post curing, printed objects are less strong
Direct energy deposition	Materials are fused by melting them using thermal energy.	LMD, DALM, DMD, LDD	Metals	Used to produce high-quality and functional parts	Limited material can be used, poor surface finish and accuracy
Materials extrusion	A certain size of filament is made to pass through feeding roller, heater, and nozzle to print the object layer by layer.	FDM	Polymer-based materials	Low machine cost, easy handling of materials, no post curing	Poor surface finish and accuracy, slow processing for large parts, anisotropic nature of printed parts
Material jetting	Droplets of build material are selectively deposited.	MJM	Polymers, waxes	Single part can be produced from multiple materials having different characteristics and properties, very precise and smooth surface finish	Requires support materials, expensive technology
Powder bed fusion	Thermal energy is used to fuse the powder bed region.	EBM, SLS, SLM, DMLS, SHS	Metals, polymers	Does not require support structure, can produce complex parts	Poor surface finish
Sheet lamination	Sheets of material are bonded to form an object.	LOM, UC	Metals, paper	Low cost, ease of material handling, high speed	Limited material use, requires postprocessing
Vat photopolymerization	Liquid photopolymer in a vat is selectively cured by light-activated or ultraviolet polymerization.	SLA, DLP	Photopolymers	Good surface finish, can fabricate the very accurate and complex design	Support structure needed, requires postcuring and postprocessing

*BJ, binder jetting; PBIH, powder bed and inkjet head; PP, plaster based 3D printing; LMD, laser metal deposition; DALM, direct additive laser manufacturing; DMD, direct metal deposition; LDD, laser direct deposition; FDM, fused deposition modeling; MJM, multijet modeling; EBM, electron beam melting; SLS, selective laser sintering; SLM, selective laser melting; DMLS, direct metal laser sintering; SHS, selective heat sintering; LOM, laminated object manufacturing; UC, ultrasonic consolidation; SLA, stereolithography (apparatus); DLP, digital light processing*.

Among different types of the additive manufacturing process, FDM or fused filament fabrication (FFF) is a rapid, versatile, low-cost, and mostly used 3D printing technique that fabricates a complex-shaped part easily and promptly (Tran et al., 2017). Relatively low printer cost and the requirement of little technical knowledge to run the machine make this technique the most popular among all techniques (Ecker et al., 2017). In FDM, a well-shaped thermoplastic filament is heated into the semiliquid state, which is extruded through the nozzle and deposited layer by layer onto the build platform. The deposited layers fuse together and solidify to form the required final object (Ngo et al., 2018; Xu et al., 2018b).

Most commonly used thermoplastic materials are polylactic acid (PLA) (Valerga et al., 2018; Liu Z. et al., 2019), poly(ε-caprolactone) (PCL) (Chim et al., 2006; Goyanes et al., 2016; Tran et al., 2017), ethylene vinyl acetate (EVA) (Kumar et al., 2018), polyamides (Terekhina et al., 2019), and acrylonitrile butadiene styrene (ABS) (McLouth et al., 2017; Harris et al., 2019). Chaunier et al. (2018) mentioned that the polymers that have a processing temperature higher than the transition temperature and lower than the degradation temperature with a rigidity of ≥1 GPa can be used for FDM application. The major drawbacks of the FDM technique are poor parts and anisotropic mechanical properties, poor surface quality, high hygroscopic sensitivity, the need for supports, and limited thermoplastic material as feedstock. However, drawbacks like poor mechanical properties and surface finish can be improved by changing several processing parameters such as build direction, printing temperature, feed rate, layer thickness, raster angle, raster width, infill density, and pattern (Le Duigou et al., 2016; Chiulan et al., 2017; Gonzalez Ausejo et al., 2018a,b). Therefore, researchers are focusing on different printing parameters to minimize the shortcomings of this method.

Most of the materials used as filaments for FDM are not environment-friendly since they are petroleum-based and could release toxic substances during the printing process, which has an adverse effect on health and environment (Wu and Liao, 2017a). Hence, research regarding the development of a biobased filament for FDM is gaining a lot of attention, which not only helps to reduce the use of petroleum-derived plastic but also reduces the cost of filaments. Many reviews were carried out on the use of biomaterials for 3D printing (Li et al., 2014; Chia and Wu, 2015; Chiulan et al., 2017; Tappa and Jammalamadaka, 2018; Xu et al., 2018b; Mazzanti et al., 2019), and most of them were focused on the biomedical applications (Li et al., 2014; Chia and Wu, 2015; Chiulan et al., 2017; Tappa and Jammalamadaka, 2018; Xu et al., 2018b). Most of the papers on the use of biomaterials for 3D printing included different 3D printing techniques. In this review paper, the focus is on the use of different biomaterials for 3D printing by the FDM technique.

## Poly-Lactic Acid (PLA)

PLA is the most commonly used bioplastic (Chiulan et al., 2017), derived from the starch of agricultural plants such as corn, sugarcane, sugar beets, and wheat (Gordobil et al., 2014; Cuiffo et al., 2017). PLA is one of the most studied thermoplastic aliphatic polyesters formed from ring-opening polymerization of lactide or polycondensation of lactic acid monomer (Chiulan et al., 2017; Xu et al., 2018a). PLA can be found in semicrystalline or amorphous grade. Pure poly (l-lactic acid) (PLLA) or poly (D-lactic acid) is semicrystalline, whereas PLA with 50–93% L-lactic acid is amorphous. Amorphous PLA exhibits better processability but poor mechanical properties as compared to crystalline (Chiulan et al., 2017). PLA is biodegradable, biocompatible, and user-friendly and can be easily processed with no toxic fumes (Gkartzou et al., 2017; Xu et al., 2018a). It is found to be used for packaging and fabrication of several biomedical devices such as orthopedic implants, drug delivery systems, surgical sutures, and tissue engineering scaffolds (Chiulan et al., 2017; Xu et al., 2018a). Properties of PLA such as low glass transition temperature (T_g_ = 60°C−65°C) and melting temperature (T_m_ = 173°C−178°C), lower coefficient of thermal expansion, and non-adherence to the printing surface make it a promising thermoplastic for 3D printing purposes (Chiulan et al., 2017; Cuiffo et al., 2017). However, low thermal stability, high degradation rate during processing, brittle in nature, low toughness, moisture sensitivity, and comparatively higher cost than standard polyolefin limit its application (Gordobil et al., 2014; Chiulan et al., 2017; Gkartzou et al., 2017; Mimini et al., 2019). The average market price of PLA pellets is US$5.5/kg, whereas that of polypropylene and high-density polyethylene is around US$1.6 and US$1.7/kg, respectively (Vandi et al., 2018; Woern et al., 2018).

### 3D Printing of PLA

Jo et al. (2018) used a PLA filament to 3D-print objects and investigate the effect of layer thickness, externally applied heat, and pressure in an FDM printed 3D object. The authors found that layer thickness directly affects the mechanical properties of the printed object, and these properties could be improved on thermal heating. The authors also noticed that on heating the printed object having small layer thickness, tensile strength and elastic modulus were enhanced. This was due to the improvement in the bond between raster to raster and layer to layer. Applying higher external pressure had a similar improvement in the tensile strength and modulus of the printed object. Further, Jo et al. (2018) mentioned that controlling layer thickness, external heat, and pressure helped in reducing the void in the internal structure of the printed object and creating an object of better finish and improved mechanical properties. Similarly, (Rajpurohit and Dave, 2018) studied the effect of raster angle, layer height, and raster width on the tensile properties of FDM printed PLA parts where they found the highest tensile strength at 0° raster angle. Those samples that had lower layer height exhibited higher tensile strength because of the larger bonding area. The authors also observed higher tensile strength of a sample having higher raster width to a certain extent, but after that, it decreased due to the void formation, which helped in crack initiation and propagation. Other authors such as Yang et al. (2019) used FDM PLA printed parts to investigate the effect of nozzle diameter, liquefier temperature, extrusion velocity, filling velocity, and layer thickness on tensile strength, surface roughness, and build time of printed parts. Results obtained indicated that nozzle diameter and layer thickness are the most influencing factors on tensile strength, surface roughness, and build time of printed parts. The authors also found that with a larger nozzle diameter, high extrusion, filling velocity, and larger layer thickness, the tensile strength and surface roughness of printed parts increased noticeably, whereas there was less effect of liquefier temperature and extrusion velocity on surface roughness. Yang et al. (2019) have further noted the reduction in build time with increment in the nozzle diameter, filling velocity, and layer thickness. Furthermore, Alafaghani et al. (2017) looked at the effect of process parameters such as building direction, printing speed, extrusion temperature, layer height, infill percent, and infill patterns on the mechanical properties and dimensional accuracy of FDM printed PLA specimens. They concluded that building direction, extrusion temperature, and layer height were more influencing parameters compared to infill percentage, infill pattern, and printing speed on dimensional accuracy and mechanical properties. For the 3D print parts of higher dimensional accuracy, the direction of the part should be parallel to the layer orientation instead of the building orientation, accompanied by lower layer height and extrusion temperature. Crystallinity, thermal resistance, modulus, and strength of the FDM printed PLA sample could also be increased by increasing the bed temperature (Benwood et al., 2018). Benwood et al. (2018) mentioned that in order to maximize the bond strength between deposited layers, bed temperature needs to be above the glass transition temperature.

Another group, Afrose et al. (2016), studied the effect of build orientation on the fatigue behavior of PLA parts made by the FDM method. The parts that had X-build orientation exhibited higher tensile strength than Y- and 45°-build orientation under static loading. Under tensile loading, fatigue life was higher for the PLA specimen with 45°-build orientation and higher ability to store strain energy by part.

On comparing 3D printed PLA with injection molded PLA in terms of the mechanical response, Song et al. (2017) found that 3D printed specimen had improved toughness because of its layered and filamentous nature. Additionally, the 3D printed specimen had increased crystallinity and reduced ductility.

From all these papers related to FDM printing of PLA, it was found that research with PLA was mostly done to investigate the influence of process parameters, part orientation, and environment on FDM printing. Some parameters such as layer thickness, build direction, raster angle, raster width, infill density, extrusion temperature, and bed temperature strongly affected the mechanical properties, whereas extrusion speed, printing speed, and infill pattern had no significant effect in the mechanical properties. Besides this, the 3D printing machine and software components equally play an important role in the mechanical properties of printed parts. In all these researches, different types of printers were used, which were compatible with different slicing software and process condition. Therefore, it will be unfair to generalize and conclude based on their results. For generalization and comparison among different research scenarios, a standard set of conditions and parameters need to be developed for FDM printers and FDM printed test specimens.

Table 2 represents the summary of the effects of different printing parameters on the mechanical properties of FDM printed PLA specimens.

**Table 2:** Effect of printing parameters on mechanical properties of FDM printed PLA specimen.

**Filament diameter**	**3D printer used**	**Constant parameters**	**Variable parameters**	**Mechanical properties**	**Reference**
	Open-source FDM printer	Extruder temperature −210°C Bed temperature −70°C Speed − 50 mm/s Infill% − 100%	Layer height (μm) − 100, 150, 200, 250, 300 Raster width (μm) − 400, 500, 600, 700 Raster angle − 0°, 30^o^, 45^o^, 60^o^, 90^o^	At constant raster width, TS ↓ with the ↑ in raster angle. TS ↓ with ↑ in layer height. TS ↑ with ↑ in raster width up to 600 and then ↓.	Rajpurohit and Dave, 2018
1.75 mm	Raise3D N2 plus	Build orientation 0° Infill − 100%	Nozzle diameter (mm) − 0.2, 0.4, 0.6 Temperature (°C) − 200, 215, 230 Extrusion velocity (mm/s) − 20, 25, 30 Filling velocity (mm/s) − 20, 30, 40 Layer thickness (mm) − 0.1, 0.2, 0.3	↑ in nozzle diameter, extrusion temperature, filling velocity, and layer thickness led to ↑ in TS and surface roughness. Extrusion velocity had no effect on TS and surface roughness.	Yang et al., 2019
1.75 mm	Makerbot Replicator 2X 3D printer		Build direction—X, Y, Z Infill percent − 100, 20, 50, 80 Print speed (mm/s) − 90, 70, 120, 170 Extrusion temperature (°C) − 185, 175, 180, 205 Layer height (mm) − 0.3, 0.1, 0.25, 0.4 Infill pattern—diamond F, diamond, linear, hexagonal	Maximum mechanical properties for Z build direction and minimum for X build direction. Printing speed and infill pattern did not have significant effect in mechanical properties. Mechanical properties were improved with ↑ in extrusion temperature and layer height up to a certain limit.	Alafaghani et al., 2017
2.85 mm	LulzbotTaz 6 3D printer	Print speed − 50 mm/s Travel speed − 200 mm/s Fill density − 100%	Melt temperature (°C) − 190, 200, 210, 220, 230 Bed temperature (°C) − 45, 60, 75, 90, 105 Raster angle − 45° /45°, 30°/60°, 15°/75°, 0°/90°	↑ in mechanical properties with ↑ in bed temperature. Optimal mechanical properties were observed at 45°/45° raster angle.	Benwood et al., 2018
	Cube-2 3D printer		Build direction—X, Y, and 45°	Specimen printed in X direction exhibited maximum TS and that in Y direction showed lowest. Specimen having Y build orientation displayed maximum ductility as compared to the other two. Specimen in X build direction exhibited lowest fatigue life, whereas that having 45^o^ build orientation displayed maximum fatigue life.	Afrose et al., 2016

*TS—tensile strength, E—elastic modulus, IS—impact strength, ↑–increase, ↓–decrease*.

### 3D Printing of PLA Composites

Production of PLA requires precise reaction conditions such as temperature and pressure, which accounts for higher energy consumption. In addition to that, corn-based PLA has led to increasing concern over food crisis. Adding fillers to PLA will not only decrease the amount of PLA usage and address the concern to the food crisis but also reduce the cost as compared to the use of neat PLA. Poor thermal and mechanical properties of PLA limit it for many engineering applications (Nguyen et al., 2018a). Chiulan et al. (2017) also mentioned that PLA is not able to mimic nature (e.g., native bone architecture, cell colonization) properly. Therefore, to widen its applicability for both engineering and biomedical applications, it needs to be mixed with fillers.

Tao et al. (2017) developed a composite filament of PLA and 5 wt% of wood flour (WF) of particle size 14 μm for printing a 3D object by the FDM technique. The object printed from a composite filament appeared like that of the wooden object as compared to that made from a pure PLA filament. Due to the hydrophilic nature of WF and the hydrophobic nature of PLA, there was a poor interfacial bond, which resulted in clear gaps between the PLA and WF interface. Moreover, the particle size of wood powder or any other material to be blended with PLA should be ultrafine in order to prevent nozzle blocking during printing (Wimmer et al., 2015). Also, the properties of wood particle and its compatibility with thermoplastic polymer should be taken into consideration as they affect the properties of the wood polymer composite filament (Kariz et al., 2018). Additionally, there will be variation in the properties of the composite filament with variation in wood content. Kariz et al. (2018) found that increasing wood concentration resulted in decreased filament density. There was a slight increase in tensile strength with a 10% increase in wood content, but the further increment of wood content led to decreased tensile strength. Guo et al. (2018) got the similar result of poor mechanical properties with an increase in the poplar WF content in PLA. They explored different toughening agents for the PLA/WF composite filament, namely thermoplastic polyurethane (TPU), polycaprolactone (PCL), and poly(ethylene-co-octene) (POE), and found that among all, TPU relatively showed better compatibility with the PLA/WF composite. They also mentioned that impact strength, tensile strength, ductility, complex viscosity, and storage modulus of the composite were increased. Ayrilmis (2018) studied the effect of layer thickness on surface roughness and wettability of 3D printed object prepared from the PLA/WF filament and found a direct impact of layer thickness on both properties. It was observed that with the increase in layer thickness, both surface roughness and wettability increased for the 3D printed object from the PLA/WF filament.

Another group, Daver et al. (2018), successfully developed a composite filament of PLA and 5 wt% cork for FDM application. It was found that with the increase in cork content from 0 to 50 wt%, the tensile strength, elastic modulus, and elongation at break were decreased from 60 to 10 MPa, 3.35 to 1 GPa, and 1.53 to 0.15%, respectively. On the other hand, impact strength decreased initially and increased on further addition of cork. With the further addition of a biodegradable plasticizer named tributyl citrate (TBC), ductility of the composite was enhanced but their strength and modulus were decreased. On the comparison between a 3D printed specimen and a compression molded specimen made from a cork–PLA composite, it was found that the 3D printed specimen had a higher elongation at break than the latter. However, its elastic modulus and tensile strength were lower than those of the compression molded specimen.

Murphy and Collins (2018) worked on the development of fully degradable biocomposite filaments for FDM application from microcrystalline cellulose (MCC) and PLA. They found that with the addition of MCC from 1 to 3 wt%, the crystallinity of PLA and the storage modulus of the biocomposite were increased. Dong et al. (2017) extruded a composite filament for FDM application from neat PLA and poly(lactic acid) grafted cellulose nanofibers (PLA-g-CNFs), where PLA-g-CNFs were prepared by grafting L-lactide monomers on cellulose nanofibers (CNFs). The composite filament had improved storage modulus due to the incorporation of PLA-g-CNFs. Highest elastic modulus and tensile strength of 2,800 and 39 MPa, respectively, were obtained at 3 wt% of PLA-g-CNFs. The authors also revealed that on annealing the extruded composite filaments, the crystallinity of the composite filament was increased, which led to enhancement in mechanical properties.

Xu et al. (2018a) applied a solvent blending approach to uniformly blend galactoglucomannan (GGM), a hemicellulose type found in softwood, and PLA. This blended composite was used for making a filament for FDM application and showed higher storage modulus and decreased thermal stability than neat PLA. PLA with up to 20 wt% GGM exhibited a flexural modulus similar to that of PLA around 3.2 GPa.

Lignin is the second most abundant plant-based polymer that is obtained as the by-product from pulp and paper industries and bioethanol industries. With an objective of utilizing a different type of lignin to produce a value-added product, Mimini et al. (2019) used it and compared the compatibility of kraft lignin (KL), organosolv lignin (OSL), and lignosulfonate (LS) with PLA to produce biocomposite filaments for 3D printing. The mechanical behavior was poor for the KL/PLA specimen, whereas the OSL/PLA specimen showed higher compatibility as compared to others. KL/PLA and OSL/PLA composites exhibited better thermal resistance as compared to LS. There was no improvement in the flexure strength of PLA with the addition of any of that lignin. Similarly, Gkartzou et al. (2017) conducted a research using PLA with low-cost kraft lignin, where it was found that the addition of lignin content led the blend sample to become heterogeneous that resulted in increased surface roughness and affected thermal stability. In fact, there was an increase in PLA's brittleness because of lignin aggregates, whereas no adverse effect was seen on the modulus of elasticity. The authors also revealed that with the addition of lignin from 0 to 15 wt%, tensile strength and elongation at break of the PLA/lignin composite decreased from 56 to 41 MPa and 4.57 to 1.88%, respectively.

Domínguez-Robles et al. (2019) prepared 3D printable filaments having antioxidant properties using PLA and (3 wt%) lignin. Materials printed from these filaments could be used in different healthcare applications like wound healing. Filaments were prepared by extruding PLA pellets coated with lignin and castor oil. Because of the incorporation of lignin, Domínguez-Robles et al. (2019) found an increase in a maximum load before fracture and higher wettability.

A composite filament for 3D printing was developed using PLA and biocarbon, derived from the pyrolysis of wheat stems, and processing and wear behavior of the printed specimen were studied (Welzel et al., 2018). It was found that specimens fabricated from PLA and 30 vol% biocarbon had less wear volume and a high coefficient of friction with fewer fluctuations. They also mentioned that with the increase in vol% of biocarbon in the composite, there was an increase in voids in printed samples and difficulty in printing due to nozzle clogging.

Ou-Yang et al. (2018) prepared the filament of PLA/poly(butylene succinate) (PBS) blend for 3D printing where they observed that all blends had excellent processing properties. The blends having PLA of more than 40 wt% had smooth printing without any distortion or detachment from the printing surface and higher tensile strength, modulus, and melt viscosity, and showed better suitability for FDM. The maximum tensile strength of the printed sample was 21 MPa for blend composition PBS/PLA (40/60).

The study of the effect of 3D printing direction in the thermal and mechanical properties of a specimen printed from a PLA/polyhydroxyalkanoate (PHA) composite filament revealed that a vertically printed specimen had better mechanical properties than a horizontally printed specimen (Gonzalez Ausejo et al., 2018b). The horizontally printed specimen had longer disintegration time than the vertical specimen, and degradation was more distinct at 50°C. Based on the observation, the contact time of the specimen with a printing platform influenced their crystalline phase; however, an additional study concluded that not only the specimen's contact time on the printing surface affected crystallinity, but also the size of the specimen played a vital role (Gonzalez Ausejo et al., 2018a). During the printing process, the specimen having a smaller surface area had increased crystalline phase.

Antoniac et al. (2019) extruded the PLA + Mg + vitamin E (α-tocopherol) composed filament of 1.75-mm diameter for manufacturing test samples using the FDM process. They found good integration between Mg and the PLA matrix due to the use of vitamin E during material preparation. However, according to the obtained results, the authors were not able to guarantee the uniform distribution of Mg with the PLA matrix.

Prashantha and Roger (2017) studied the 3D printed specimen made up of PLA/graphene nanocomposites containing 10 wt% graphene in the PLA matrix. It was detected that the specimen printed from these filaments by the FDM technique had improved thermal and mechanical properties compared to the object printed from neat PLA filaments. The addition of 10 wt% graphene in PLA increased the modulus and strength of PLA from 1,827 to 2,454 MPa and 31.6 to 40.2 MPa, respectively. Furthermore, the uniform distribution of graphene in the PLA matrix was also found from the scanning electron microscopy of the printed object.

Ferreira et al. (2017) compared 3D printed PLA and PLA with carbon fibers (CFs) (reinforced with 15 wt% short CFs of length about 60 μm) and found that the reinforced material had increased stiffness in the direction of printing due to the alignment of short CFs in the printing direction. However, they found that on adding short CFs, printed samples turned out to be brittle. Poor adhesion between PLA and CFs was observed possibly due to the shorter length of CFs.

Rasselet et al. (2019) found improved tensile properties and ductile behavior of the 3D printed object of the PLA/polyamide 11 (PA11) blend, with 3 wt% incorporation of Joncryl, a multifunctionalized epoxide. From the results of the SEM of the tensile fracture surface, they observed the improved interfacial adhesion, which was due to Joncryl. They observed the maximum tensile strength and elongation at break of 58.8 MPa at 2 wt% Joncryl content and 9.8% at 3 wt% Joncryl content, respectively. 3D printed samples from the PLA/PA11 composite showed a brittle nature compared to that of an injected sample. This was because of poor adhesion and porosity between the deposited layers, whereas the elastic modulus was higher for an FDM printed specimen as compared to an injection molded specimen.

To reduce the excess use and high cost of PLA, and to widen its applicability in a diverse field, the trend of using different fillers with PLA to develop biocomposite filaments has increased. Among all these fillers, WF is the extensively studied and the biomaterial used to develop composite filaments. Besides WF, CNF and lignin are two other promising biobased materials that have an abundant and sustainable source and that need further research and development. Filler content, filler size, and printing parameters had high influence in the properties of the printed parts. Therefore, it is necessary to determine the application of the prepared composites first, and the influencing parameters should be set up in an optimal way according to the required properties. Investigation of several additives should be done to improve the composites' properties so that they will be able to replace widely used petroleum-based composite filaments.

Table 3 provides a brief summary of the effects of different fillers and their concentration on mechanical, thermal, and morphological properties of PLA composites.

**Table 3:** Summary of mechanical, thermal, and morphological properties of PLA composites.

**Trade name**	**Filler**	**Filler content**	**Mechanical**	**Thermal**	**Morphology**	**Reference**
Ingeo 4032D	WF (14 μm)	5 wt%	↑ in TS with ↑ of WF in 0-1.5% strain range. ↑ in E of PLA/WF composite.	↓ in T_g_ and T_cc_ of PLA with ↑ in WF.	Poor interfacial bonding between PLA and WF.	Tao et al., 2017
Ingeo 2003D	Beech wood (mesh size 0.237 mm)	10, 20, 30, 40, 50 wt%	↑ in TS and E up to 10 wt% and 20 wt% WF, respectively, and then ↓ on further ↑ WF.	No change in T_g_.	With ↑ of WF, printed surface became rougher with more pores and visible agglomeration of wood particles.	Kariz et al., 2018
Ingeo 4032D	Poplar WF (100 meshes) TPU PCL POE	10 wt% 10 wt% 10 wt% 10 wt%	↓ in mechanical properties with ↑ in WF. ↑ in IS and TS on adding TPU in PLA/WF composite. Incorporating PCL and POE to composite ↓ its IS.	No change in crystallinity of PLA with ↑ in WF. Maximum ↑ in crystallinity of PLA/WF with POE, making it brittle. No change in T_g_ of PLA with addition of TPU.	Poor interfacial interaction between WF and PLA. Adding TPU improved the interfacial interaction between WF and PLA, whereas adding POE made fracture surface of composite rougher.	Guo et al., 2018
Commercial PLA/WF filament	WF	30 wt%			↑ in surface roughness with ↑ in layer height.	Ayrilmis, 2018
Ingeo 4032D	Cork powder	5, 10, 15, 20, 25, 30, 50 wt%	↓ in TS, E, and elongation at break of PLA/cork composite with ↑ in cork content.	Crystallinity of PLA was enhanced with ↑ in cork content.		Daver et al., 2018
Ingeo 4032D	PLA-g-CNFs	1, 3, 5 wt%	Maximum TS and E at 3 wt% PLA-g-CNFs.	T_g_ of PLA was unaffected while crystallinity was ↑ by 7.9% on 3 wt% addition of PLA-g-CNFs.	Homogenous distribution of 5 wt% PLA-g-CNFs in the PLA matrix.	Dong et al., 2017
Ingeo 4043D	GGM	1, 5, 10, 15, 20, 25 wt%	Similar flexural modulus as that of PLA up to 20 wt% addition of GGM.	T_g_ of PLA/GGM blend was not changed significantly. ↓ in thermal stability upon addition of GGM to the PLA matrix.	Agglomeration of GGM was seen in both filaments and printed parts. PLA changed from continuous phase to spongy structure on adding GGM above 20 wt%.	Xu et al., 2018a
Ingeo 2002D	PBS	20, 40, 60, 80 wt%	Maximum ductility was observed in PBS/PLA (80:20 wt%) blend. TS of printed bar was maximum for PBS/PLA blend of composition 40:60.	With PBS content above 60 wt%, recrystallization of PBS was seen during heating. Degree of crystallinity of FDM printed parts was higher than raw filaments.	With PBS content less than 60%, PLA/PBS blend had no visible distortion. Serious distortion was seen with PBS more than 80%.	Ou-Yang et al., 2018
	Mg Vitamin E	6 g of 100 μm 4 g of 125 μm 2 g		T_g_ was reduced due to Mg.	Incorporation of vitamin E enhanced the integration of Mg particles in the PLA matrix. No agglomeration of filler in the polymer matrix.	Antoniac et al., 2019
Commercial filament	Graphene	10 wt%	↑ in TS and E on incorporating 10 wt% graphene.	On adding 10 wt% graphene, T_g_ of PLA was ↑ by 4°C.	Good interlayer adhesion. Homogenous dispersion of graphene nanocomposite in the PLA matrix.	Prashantha and Roger, 2017
Ingeo 4043D	Chopped short CFs	15 wt%	On adding 15 wt% of CFs, E of PLA was ↑ whereas TS was ↓.		CFs were aligned along the filament length as well as along the printed test specimen. Due to fiber pull out during failure of test specimen, voids were found.	Ferreira et al., 2017
Ingeo 3251D	Polyamide 11 Joncryl ADR- 4368	20 wt% 0–3 wt%	PLA/PA11 (80/20) composite showed similar mechanical behavior as that of neat PLA. PLA/PA11 composite showed highest elongation at break and TS 3 and 2 wt% Joncryl content, respectively.	↑ in degree of crystallinity of PLA on adding PA11. ↓ in crystallinity of PLA/PA11 on 3 wt% addition of Joncryl.	PA11 dispersed phased were present in both the filament and the 3D printed specimen. Poor interfacial adhesion with Joncryl content ≤1.5 wt%, which led to brittle failure.	Rasselet et al., 2019
Ingeo 3D850	Lignin Castor oil	0.5, 1, 2 and 3 wt% 40 μl	Maximum load before failure ↓ with ↑ of lignin from 0 to 2 wt% and then ↑ at 3 wt%.	No effect on melting behavior on adding lignin. On adding 2 and 3 wt% lignin, T_g_ of PLA ↓.		Domínguez-Robles et al., 2019
Ingeo 2003D	KL	5, 10, 15, 20 wt%	Elongation at break and TS of PLA/lignin composite ↓ on ↑ lignin. No effect on E of PLA/lignin composite with addition of lignin.	Double melting behavior of PLA was furthermore enhanced on addition of lignin. No significant difference in T_g_ and T_m_ was observed at various lignin concentrations.	On adding 5 wt% lignin, uniform distribution of <20 μm sized lignin aggregates in the PLA matrix was observed. On adding 20 wt% lignin, aggregation concentration ↑ due to coalescence of lignin particles.	Gkartzou et al., 2017
Ingeo 4043D	KL OL LS	5, 10, 15 wt%	No improvement in flexural strength on incorporating any of that lignin. ↓ in IS with ↑ in lignin content.	LS-PLA composite had highest degree of decomposition as compared to OSL and KL. On adding 15 wt% KL, OSL, and LS, T_g_ and T_m_ of PLA ↓.	Particle size of OSL lignin was 200 times smaller than KL and LS. Cavities were observed between PLA and LS particle, which became homogenous and smaller with the ↑ in LS content to 15 wt%.	Mimini et al., 2019

*T_cc_–cold crystallization temperature. ↑ increase and ↓ decrease*.

## Polyhydroxyalkanoate (PHA)

PHA is one of the natural polymers derived from the polymerization of microorganisms by eicosanoic acid (Wu and Liao, 2017a). According to Chiulan et al. (2017), under some unbalanced growing conditions such as the low concentration of nitrogen, phosphorus, oxygen, or magnesium and an excess carbon, some bacteria get synthesized into inclusion to form PHA. Despite having promising properties such as biodegradability, biocompatibility, processability, and comparable mechanical properties, higher production cost limits its applications. The average market price of PHA pellets is around US$7/kg; however, the price can be higher (above US$10/kg) for the one used for biomedical application (Vandi et al., 2018). Among different PHAs, poly(3-hydroxybutyrate) (PHB) and poly(3-hydroxybutyrate-co-3-hydroxyvalerate) (PHBV) are the most studied ones (Chiulan et al., 2017). Less carbon atom and shorter chain length of PHB result in brittle, tough nature and poor processing properties. PHBV is formed by copolymerization with hydroxyvalerate (HV) so that it possesses longer chain length and is ductile in nature (Chiulan et al., 2017; Menčík et al., 2018).

### 3D Printing of PHA Composites

Incorporating fillers in PHA to make biocomposite filaments for FDM application is definitely a way to valorize and reduce the cost of PHA (Vandi et al., 2018). Wu and Liao (2017a) compared 3D printed specimens made from a PHA/WF composite filament and from a maleic acid grafted PHA (PHA-g-MA)/WF composite. They found that the one printed from the PHA-g-MA/WF composite filament had better mechanical properties and higher quality, processability, and water resistance capacity than that of the PHA/WF composite filament. It was also revealed that with the addition of WF, there was an enhancement in antibacterial properties but a decrease in the melting temperature of the composite.

Vaidya et al. (2019) blended PHB with biorefinery lignin to form a biocomposite filament for 3D printing. They concluded that there was no reaction between lignin and PHB within the composite filament because no significant change in melting, decomposition, and crystallization temperature of PHB was observed. However, the storage modulus of PHB was decreased from 4.1 to 1.7 GPa with 20 wt% of lignin. They also revealed that lignin in the composite helped to improve interlayer adhesion and reduced the deformation of the 3D printed object.

Menčík et al. (2018) found that incorporation of a plasticizer such as acetyl tributyl citrate and tributyl citrate enhanced the elongation at break of PHB/PLA blends by 308 and 155%, respectively. The SEM image of the 3D printed sample printed from a 60% PHB/25% PLA/15% plasticizer (acetyl tributyl citrate and tributyl citrate) filament had a smooth surface and a compact area without large holes. Wu and Liao (2017b) found improved mechanical, thermal, conductive, and antibacterial properties of 3D printing filaments developed from maleic acid grafted PHA (PHA-g-MA) and acid oxidized multiwalled carbon nanotubes (MWCNTs), which may be possibly due to the interaction between nanotubes and the PHA matrix. The tensile strength and modulus of PHA-g-MA was increased from 16 to 32 MPa and 350 to 467 MPa, respectively, with the addition of 1 wt% of MWCNTs-COOH, but decreased on further increasing the filler content. Wu et al. (2017) further worked on the development of 3D printing composite filaments using maleic anhydride-grafted polyhydroxyalkanoate (PHA-g-MA) and coupling agent-treated palm fiber (TPF). Better compatibility of TPF with PHA-g-MA led to improved tensile strength. They found that tensile strength at break and Young's modulus of the composite were increased by 7 and 65 MPa, respectively, with 20 wt% TPF content in the filament. However, there was a decrease in Young's modulus of the composite filament with an increase in the TPF content above 20 wt%, which was possibly due to an aggregation of TPF.

Research regarding FDM printing of PHA composites has increased in the last few years. WF, lignin, palm fibers, and PLA are some biomaterials that were used along with PHA for making composite filaments for 3D printing. Adding biomaterials with PHA will decrease the usage of PHA and be able to overcome one of the disadvantages of PHA, i.e., higher production cost. From the above reviews, it was found that modification in PHA by grafting maleic acid resulted in improved interaction between the PHA matrix and filler, which further improved the mechanical properties of the composites. Therefore, compatibility of different other biomaterials and different strategies of blending with PHA needs to be explored for developing PHA composite filaments. Besides this, their area of application should be determined, and properties optimization should be done accordingly.

Table 4 represents the effect of different fillers and their concentration on the mechanical, thermal, and morphological properties of PHA composites.

**Table 4:** Summary of mechanical, thermal, and morphological properties of PHA composites.

**Matrix**	**Filler**	**Filler content**	**Mechanical**	**Thermal**	**Morphology**	**Reference**
PHA PHA-g-MA	WF	10, 20, 30, 40 wt%	TS of PHA/WF composite ↓ with the ↑ in WF. TS of PHA-g-WF ↑ with the ↑ in WF.	↓ in T_m_ with ↑ in WF content in both PHA/WF and PHA-g-WF composites. T_m_ was higher for PHA/WF than PHA-g-WF at same WF content. For both composites, there was ↑ in T_g_ with the ↑ in WF content.	Uniform dispersion of WF in PHA/WF (20 wt%) composite; however, poor adhesion between WF and the PHA matrix. Improved interfacial adhesion between PHA-g-MA and WF.	Wu and Liao, 2017a
PHB	Lignin (from Pinus radiate wood chips)	10, 20, 50 wt%	Storage modulus of PHB was ↓ with addition of 20 wt% of lignin.	No change in TGA profile of PHB with addition of lignin. No significant change in T_m_ and crystallinity of PHB with addition of lignin.	Filament had polymer rich surface and lignin particles in the central core.	Vaidya et al., 2019
PHA PHA-g-MA	MWCNTs MWCNTs-COOH	0.5, 1, 2, 3 wt%	Better TS and E were exhibited by PHA-g-MA/MWCNTs-COOH than PHA/MWCNTs. TS and E of PHA-g-MA/MWCNTs-COOH ↑ with addition of MWCNTs-COOH up to 1 wt% and then ↓ on higher filler content.	T_g_ ↑ with addition of MWCNTs-COOH till 1 wt% and then ↓. Addition of both types of filler led to ↓ in T_m_ of composites.	1 wt% MWCNTS-COOH content was well-dispersed in the polymer matrix; however, on increment of filler to 3 wt%, agglomerations were observed.	Wu and Liao, 2017b
PHA PHA-g-MA	PF Treated palm fiber (TPF)	10, 20, 30, 40 wt%	↓ in E and TS of PHA/PF with ↑ in PF. E of PHA-g-MA/TPF ↑ with addition of 20 wt% TPF and then ↓. TS of PHA-g-MA/TPF ↑ initially and then ↓ on adding TPF.		PF was well-dispersed in the PHA matrix, but poor adhesion between filler and polymer was observed. Better adhesion of PHA-g-MA and TPF (20 wt%).	Wu et al., 2017
PHB	PLA Plasticizer (Tributyl Citrate, Acetyl tributyl Citrate, Acetyl trihexyl Citrate, n-Butyryl tri-n-hexyl Citrate)	25 wt% 15 wt%	Tributyl citrate and acetyl tributyl citrate significantly improved elongation at break. E and TS of specimen prepared using tributyl citrate and acetyl tributyl citrate was low after 7 days of sample preparation.	Significant drop in crystallization and T_m_ of PHB/PLA blend on incorporating tributyl citrate. T_g_ of PLA was ↓ on using tributyl citrate and acetyl tributyl citrate; however, it was ↑ on adding acetyl trihexyl citrate and n-butyryl tri-n-hexyl citrate.	Object printed from composite with plasticizer tributyl citrate and acetyl tributyl citrate was well-miscible, had compact, smooth surface and smaller holes as compared to that containing remaining two.	Menčík et al., 2018

## 3D Printing of Composites of Petroleum-Based Polymers and Biofillers

Besides using a biobased polymer matrix, several researches were also done on a petroleum-based polymer matrix with biofiller. Petroleum-based polymers such as ABS, nylon, acrylic styrene acrylonitrile (ASA), and high impact polystyrene (HIPS) have better mechanical properties as compared to PLA and PHA. There are several biomaterials such as rice straw, lignin, and wood flour that are considered as by-products and have low market price. Incorporating such biofillers in these polymers will not only decrease the use of petroleum-based polymers but also increase the value of biofillers.

Dadmun et al. (2017) investigated the effect of lignin-coated cellulose nanocrystal (L-CNC) on an L-CNC/ABS composite filament for 3D printing and found that with the addition of 4 wt% of L-CNC, tensile strength increased but then decreased when further adding 10 wt%, while the tensile modulus increased up to 6 wt% L-CNC and then decreased when increasing the filler content. Additionally, 3D printed L-CNC/ABS nanocomposites had improved thermal stability and good dispersion and interfacial adhesion.

Osman et al. (2018) found a significant drop in tensile properties up to 10 wt% rice straw (RS) in ABS, but on further addition of rice straw in the ABS/RS composite, the drop in tensile properties was reduced. They also indicated that the reason behind the poor mechanical properties of the ABS/RS composite was porosity. With the increase in RS, porosity was increased, which led to the decline in mechanical properties.

Nguyen et al. (2018b) found that the modulus of elasticity remained comparable with the addition of lignin (40 wt%) in ABS. The problem of increased brittleness due to the addition of lignin in ABS was resolved by the addition of acrylonitrile butadiene rubber (NBR41, 41 mol% nitrile content). The ABS/lignin composite displayed excellent plasticity and prominent increase in tensile strength with 10 wt% addition of NBR41. Mechanical properties were further enhanced with just 10 wt% addition of CFs in the ABS/lignin/NBR41 composite. Akato et al. (2015) revealed that the addition of 10 wt% PEO (polyethylene oxide) in the ABS/lignin (30 wt%) composite showed similar properties to that of neat ABS. Nguyen et al. (2018a) performed research on a nylon 12/hardwood lignin (6:4) composite, where they observed a significant increase in the material's strength and stiffness with the addition of CFs.

Akato et al. (2015) revealed that the use of kraft lignin simulates a strong olfactory response, which could be detrimental for a commercial approach. They performed further experiments using organosolv (Alcell) lignin instead of kraft lignin and found that unpleasant odor was eliminated because of the absence of sulfur. Finally, they concluded that all lignin-containing hydroxyl groups such as organosolv lignin, soda pulped lignin, and lignin from biorefinery residues can be used for composite formation. Additionally, Nguyen et al. (2018a) mentioned that organosolv hardwood lignin offers good thermal processing and good printability characteristics in contradiction to kraft softwood lignin, which has higher viscosity. Tran et al. (2017) fabricated a biofilament for FDM application from poly(ε-caprolactone) (PCL), a biodegradable polymer and cocoa shell waste. Homogenous distribution of cocoa shell waste in a PCL matrix was observed from SEM, and there was no significant difference in crystallinity and stiffness between a PCL/cocoa shell biofilament and a pure PCL filament. According to Tran et al. (2017), 3D printed specimens from these biofilaments had better layer adhesion and fine resolution.

Since chain branched amylopectin exhibits poor processability, Kuo et al. (2016) debranched starch with α-isoamylase and used glycerol and water as a plasticizer to prepare thermoplastic starch (TPS), which was blended with ABS to make a filament for 3D printing. The physical properties of only TPS/ABS (30/70 wt%) did not meet the requirement of the polymeric material used for 3D printing. However, adding a compatibilizer [styrene maleic anhydride copolymer (SMA)] improved heat stability, flowability, and mechanical properties. When a 2 wt% impact modifier [methylmethacrylate butadiene styrene (MBS)] was further added to a TPS/ABS/SMA composite, the composite exhibited better physical properties than commercial ABS. However, the heat distortion temperature was not satisfactory. Further, Kuo et al. (2016) added TiO_2_, which improved thermal properties. On replacing TiO_2_ with carbon black, they found further improvement in thermal stability, flowability, and mechanical properties. Filaments made up of TPS/ABS/SMA/MBS/TiO_2_ and TPS/ABS/SMA/MBS/CB both had lower volatile organic compound emission (VOC) than the commercial ABS.

Application of biomaterials to develop biocomposite filaments for FDM is an emerging field. On reviewing several papers based on the development of composite filaments using petroleum-based polymer and biofiller, it was found that there was reduction in the mechanical properties of composites on adding a higher amount of biofiller. Several strategies such as adding plasticizers, compatibilizer, and CFs were implemented to improve the composite's properties comparable or better than original petroleum-based polymers. Biomaterials that have abundant and sustainable sources should be examined and used as a filler in the polymer matrix for developing filaments. Besides ABS, a widely studied polymer for preparing biocomposite filaments, ample studies of compatibility of different biofillers with other petroleum-based polymers such as HIPS, ASA, nylon, and PCL should also be done. At the same time, focus toward tuning composites' properties according to their application should be increased in order to transfer lab-scale experiments to mass production and commercialization.

Table 5 represents a brief summary of the usage of different petroleum-based polymers with biofillers. It also presents the effect of biofiller content on the mechanical, thermal, and morphological properties of composites.

**Table 5:** Summary of mechanical, thermal, and morphological properties of petroleum-based polymers with biofillers.

**Matrix**	**Filler**	**Filler content**	**Mechanical**	**Thermal**	**Morphology**	**Reference**
ABS	L-CNC	2, 4, 6, 8, 10 wt%	↑ in TS up to 4 wt% addition of L-CNC but rapid ↓ upon further addition till 10 wt%. ↑ in E up to 6 wt% L-CNC and then ↓ afterward.	Reduction of initial degradation temperature with addition of L-CNC. At high temperature, ↑ in thermal stability with ↑ in L-CNC.	Uniform distribution of L-CNC in the ABS matrix. Presence of pores in 3D printed part from ABS/ L-CNC composite filament. ↑ in pore diameter to >30 μm with ↑ in L-CNC content above 6 wt%.	Dadmun et al., 2017
ABS	RS	5, 10, 15, 20 wt%	↓ in TS and E with addition of RS. With ↑ in RS content, flexure modulus and strength ↓; however, both ↑ at 15 wt% RS.		Printed parts looked like wood, got darker and porosity ↑ with addition of RS.	Osman et al., 2018
ABS	Lignin NBR41 CFs (1/8 inch)	40 wt%10 wt%10 wt%	Incorporation of 40 wt% lignin in ABS ↓ TS of composite, which was improved on adding NBR41 and CFs. E of ABS was ↓ on adding lignin and NBR41 while CFs ↑ E.	T_g_ of composite was ↓ on adding lignin, NBR42, and CFs.	Well-dispersed phase separated lignin was seen. Percolation of CFs was observed in composite. On adding 10 wt% CFs to ABS/lignin/NBR41 composite improved the interlayer adhesion between two printing layers.	Nguyen et al., 2018b
ABS	Lignin PEO CFs	10, 20, 30 wt% 10 wt% (relative to lignin amount) 20 vol%	No effect was observed on E due to PEO. PEO ↑ the elongation at failure. ↑ in TS with the addition of CFs in ABS/lignin/PEO (70/27/3) composite.	PEO retarded early decomposition of lignin. Presence of PEO led to ↑ in degradation peak temperature. PEO lowered the T_g_ of ABS.	Enhancement in interfacial adhesion between the ABS matrix and lignin particle with addition of PEO. ↓ in lignin domain size from 300–1,000 nm to 200–500 nm in the ABS matrix with incorporation of PEO.	Akato et al., 2015
Nylon 12	HW lignin CFs	40–60 wt% 4–16 wt%	Addition of 40 wt% lignin to nylon 12 matrix, led to ↑ in E while TS was nearly same as neat nylon 12. ↑ in TS and E with the addition of 12 wt% CFs in nylon 12/lignin (6:4) composite.	Noticeable ↓ in T_m_ and recrystallization temperature due to CFs and lignin. ↑ in thermal conductivity of nylon 12/lignin (6:4) composite with addition of CFs.	CFs were well-dispersed in the polymer matrix. Spherical aggregated lignin phases were seen in the polymer matrix.	Nguyen et al., 2018a
PCL	CSW (50 μm)	10, 20, 30, 40, 50 wt%	On addition of CSW to 30 wt%, E of filament ↑ and then ↓. Tensile strain at break ↓ with addition of CSW.	Minimal change in thermal properties of te PCL matrix on blending CSW.	Uniform distribution of CSW in the PCL matrix, no clumping and clustering was observed. 3D printed specimen had good interlayer adhesion with no voids and gaps.	Tran et al., 2017

## Conclusions

From this review, it was discovered that biobased materials can be used in three different ways as a feedstock for FDM. They are as follows: (1) using biobased polymers such as PLA or PHA alone; (2) blending these biobased polymers with fillers; and (3) blending petroleum-based polymers such as ABS, nylon, and PCL with biobased fillers. Most of the researches carried out on FDM printing of PLA were focused on the study of process parameters on the mechanical properties of printed parts. Various printing parameters were altered to determine their effect and to obtain the printed parts with better mechanical properties. Among those different parameters, build direction, layer thickness, raster angle, raster width, extrusion, and bed temperature have significant effect on mechanical properties. Besides processing parameters, the printing machine is also equally responsible for determining the quality of printed parts. Regarding the polymer biocomposites, different biomaterials were discovered as fillers to develop biocomposite filaments for FDM. For instance, wood flour, CNFs, lignin, and palm fibers were commonly used fillers. Mechanical, thermal, and morphological properties of 3D printed specimens from biocomposite filaments depend on the chosen polymer matrix, the particle size and amount of filler, its method of blending with the polymer matrix, the homogeneity of filaments, and printing parameters. As the amount of filler increases, most of them results in lowered mechanical properties. Despite lots of researches and discoveries on biocomposite filaments, they are not widely accepted by industries. Problems like lower mechanical strength, poor dimensional accuracy according to design specification, and poor layer adhesion need to be overcome in order to widen the area of application of biocomposite filaments. All the influencing parameters mentioned above should be maintained in an optimal way such that printed objects have comparable or better properties than the finished products obtained from the traditional manufacturing process.

## Author Contributions

SW did the literature review, prepared the manuscript, and revised it. SA advised SW, reviewed the manuscript, and revised it for submission.

## Conflict of Interest

The authors declare that the research was conducted in the absence of any commercial or financial relationships that could be construed as a potential conflict of interest.
